# Engineering γδ T Cells: Recognizing and Activating on Their Own Way

**DOI:** 10.3389/fimmu.2022.889051

**Published:** 2022-05-06

**Authors:** Ruoyu Dong, Yixi Zhang, Haowen Xiao, Xun Zeng

**Affiliations:** ^1^ Department of Hematology, Sir Run Run Shaw Hospital, Zhejiang University School of Medicine, Hangzhou, China; ^2^ State Key Laboratory for Diagnosis and Treatment of Infectious Diseases, National Clinical Research Center for Infectious Diseases, National Medical Center for Infectious Diseases, Collaborative Innovation Center for Diagnosis and Treatment of Infectious Diseases, The First Affiliated Hospital, Zhejiang University School of Medicine, Hangzhou, China

**Keywords:** γδ T cells, engineering, stimulation, dual recognition, tumor

## Abstract

Adoptive cell therapy (ACT) with engineered T cells has emerged as a promising strategy for the treatment of malignant tumors. Among them, there is great interest in engineered γδ T cells for ACT. With both adaptive and innate immune characteristics, γδ T cells can be activated by γδ TCRs to recognize antigens in a MHC-independent manner, or by NK receptors to recognize stress-induced molecules. The dual recognition system enables γδ T cells with unique activation and cytotoxicity profiles, which should be considered for the design of engineered γδ T cells. However, the current designs of engineered γδ T cells mostly follow the strategies that used in αβ T cells, but not making good use of the specific characteristics of γδ T cells. Therefore, it is no surprising that current engineered γδ T cells in preclinical or clinical trials have limited efficacy. In this review, we summarized the patterns of antigen recognition of γδ T cells and the features of signaling pathways for the functions of γδ T cells. This review will additionally discuss current progress in engineered γδ T cells and provide insights in the design of engineered γδ T cells based on their specific characteristics.

## 1 Introduction

Immunotherapy has become one of important pillars of cancer treatment, as it can trigger and augment the power of patients’ immunity to attack malignant cells. Among immunotherapy strategies, adoptive cell therapy (ACT) with engineered T cells, such as chimeric antigen receptor (CAR)-T and T cell receptor (TCR)-T cells, has gained considerable attention (1, 2). A good example is that CAR-T therapy has advanced the furthest in clinical development and three CAR-T products (Kymriah, Yescarta, and Tecartus) have gained commercial approval in the United States.

Over the past decades, a variety of researches of γδ T cells have added to the established understanding in highlighting conspicuous roles of γδ T cells in cancers. Although some researches point the potential tumorigenic effector functions of γδ T cells (3, 4), increasing translational researches have shown great interest in the therapeutic use of certain subsets of γδ T cells, especially engineered γδ T cells. In fact, the momentum of engineered γδ T cell therapy may have been generated, as U.S. Food and Drug Administration (FDA) has cleared investigational new drug application for ADI-001 that comprises CD22-allogenic γδ CAR-T cell therapy in 2020.

Activation of γδ T cells is in a TCR-dependent process as similar as that of αβ T cells, yet in independence of major histocompatibility complex (MHC). In addition to γδ TCR signals, γδ T cells mediate multiple responses *via* receptor-ligand interaction of innate signals, similar to NK cells. They bear a variety of NK cell receptors (NKRs) such as NKG2D and NK cytotoxicity receptors (NCRs) including NKp30, NKp44, and NKp46 (5). These receptors may fine-tune the γδ T cell activation threshold, enhance γδ T cells to recognize tumor target, prompt γδ T cells to mediate an immediate immune reaction against tumor target, and release cytotoxic granules such as perforin and granzyme B. In cancer, the down-regulation of MHC-I may prompt ‘missing-self recognition’, which unlock the binding between MHC-I and inhibitory receptors on γδ T cells, making γδ T cells unhindered to attack tumor cells in a NK-like manner (6). Dual recognition and stimulation system endows γδ T cells distinct anti-tumor effect. However, current design of engineered γδ T cells is a me-too engineered αβ T cells, such as using the same single-chain fragment variable (scFv) and co-stimulation molecules which are proved to help kill tumor cells effectively in αβ T cells but not completely confirmed in γδ T cells. This kind of design may take some advantages of γδ T cells such as GVHD absence, however, this raises the question that how to make the best use of dual recognition and stimulation system of γδ T cells to endow engineered γδ T maximum anti-tumor effect.

In this review, we provided a comprehensive and deep summary of the unique patterns of γδ T recognition and signaling pathways. Based on these underlying mechanisms, this review further discussed valuable insights in the design of engineered γδ T cells. It is promising that an intelligent design that considers the specific characteristics of γδ T cells will be beneficial for the utility of engineered γδ T cells.

## 2 γδ TCR Antigen Recognition

Like αβ T cells and B cells, γδ T cells generate their specific T cell receptors (TCRs) *via* recombination activating gene (RAG)-mediated V(D)J recombination, which contributes to the high diversity up to 10^17^ theoretically possible combinations of TCR repertoires (7). Since γδ T cells have been discovered in 1980s, what antigens γδ TCRs can recognize remains an outstanding question in this field. Nowadays, it has been known that γδ TCRs antigen recognition pattern is unrestricted by MHC. The ligands that they can recognize include self-antigens, such as MHC-like molecules, B7-like molecules, and foreign-antigens, such as haptens, virus protein, phycobiliproteins (8–12). Recent researches have shown that γδ TCRs most likely take part in complicated mechanisms that involves multiple ligands on the tumor cells, as well as the sensation of spatial and conformational changes through the γδ TCRs and potentially associated molecules.

**Figure 1:**
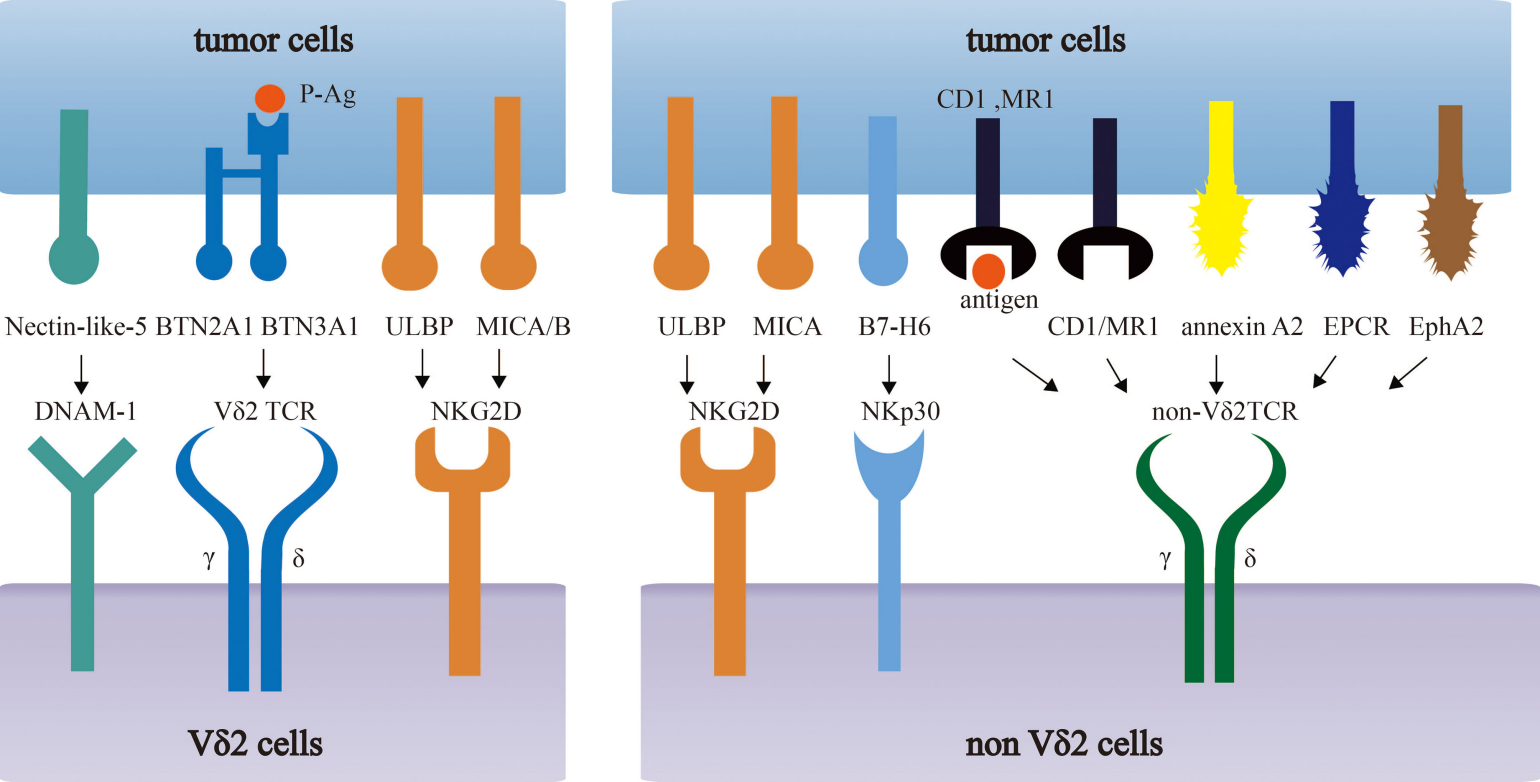
Recognition of tumor-associated antigens (TAAs) by different subsets of γδ T cells. Vδ2 T cells recognize phosphoantigens (P-Ag) modified Butyrophilin 2A1 (BTN2A1)-Butyrophilin 3A1 (BTN3A1) complex. Non-Vδ2 TCRs recognize CD1 family members and MR1 in either antigen dependent or independent manner. Besides, NK receptors (NKG2D, NKp30, DNAM-1) in γδ T cells recognize MICA/B, ULBP and B7-H6, Nectin-like-5 respectively.

The comprehensive description of antigen recognition by γδ TCRs has already been summarized in published articles (13, 14). Here, we only briefly review tumor-related antigens recognized by γδ TCRs, which are summarized in **Figure 1**.

Vγ9Vδ2 T cells recognize phosphoantigens (P-Ag) modified Butyrophilin 2A1 (BTN2A1)-Butyrophilin 3A1 (BTN3A1) complex in an MHC-independent, but TCR-dependent manner. In tumor, the dysregulation of mevalonate pathway accounts for the accumulation of phosphorylated mevalonate metabolites, such as Isopentenyl pyrophosphate (IPP) that was identified as a kind of P-Ag, and thus activate the Vγ9Vδ2 T cells (15–17). On the other hand, zoledronate (ZOL) can inhibit IPP-metabolizing enzyme, farnesyl-diphosphate-synthase (FPPS), and increase IPP level, which contributes to an enhanced IPP-induced γδ T cell activation (16, 18). ZOL has been widely used in cancer therapies such as renal cell carcinoma (RCC) and prostate cancer (19, 20). Many clinical trials have found it significantly inhibit the cancer progression and even completely cure cancers.

However, the defined molecular mechanisms of Vγ9Vδ2 T cell activation by phosphoantigens still remains to be discovered. The binding of P-Ags and the intracellular B30.2 domain of BTN3A1 leads to the conformational changes of the BTN3A1 extracellular domain, which can take part in the activation of Vγ9Vδ2^+^ TCRs (9, 21, 22). BTN2A1, a BTN molecule that is associated with BTN3A1 in extracellular and intracellular domains, directly binds to Vγ9 domain of the TCRs, potentiating Vγ9Vδ2-mediated P-Ag sensing. In addition, another Vγ9Vδ2 TCR direct interaction, mediated by BTN3A1 or an unknown ligand, is also essential in the response of Vγ9Vδ2 T cell to P-Ag (23, 24).

In addition to BTN3A molecules, the recognition of MHC or MHC-like molecules by γδ T cells has been intensively studied. γδ T cells can recognize molecules such as HLA-A24, HLA-B27 and HLA-A2 and may specifically recognize certain MHC molecules in tumor cells (13). For example, the engineered αβ T cells, which expressed Vγ5Vδ1^+^ TCRs, could be activated by HLA-A*24:02^+^ tumor cells and significantly decreased the tumor burden and enhanced survival rate of HLA-A*24:02^+^ tumor-bearing mice (25). On the other hand, some MHC-like molecules, such as MHC-related protein 1 (MR1), CD1, has a preference to specifically bind to Vδ1 TCRs in most cases (8, 26–29). Interestingly, loading different lipids may have different influences on the binding affinity of CD1d/CD1c and γδ TCRs, suggesting the loaded lipids on CD1 molecule contribute to γδ TCR antigen recognition (8, 28). MR1, another MHC-like molecule can also be recognized by γδ TCRs. γδ T cells co-cultured with MR1-transduced cells, MR1-restrict γδ TCRs transduced Jurkat-76 cell lines, can be activated with up-regulating CD69 and ERK1/2 phosphorylation (29). Although CD1/MR1-restricted NKT or αβ T cells were reported to induce specific tumor killing ability in many kinds of tumor cells (30–32), there is no evidence that γδ T cells can also lead to antitumor activity by recognizing CD1 or MR1 molecules. The role of CD1 or MR1-restricted γδ T cells in cancer immune surveillance still needs to be further studied.

Recently, more novel tumor-associated molecules that can be recognized by γδ TCRs have been revealed, including annexin A2 (33), EPCR (an MHC-like molecule) (34, 35), and ephrin type-A receptor 2 (EphA2) (36, 37). The expression of EphA2 is up-regulated in cervical cancer and colon cancer cells, which is mediated by the metabolic changes (AMP-activated protein kinase (AMPK)–dependent metabolic reprogramming) in tumor cells. γδ T cells play increasing tumor-killing ability by recognizing EphA2. This ability can be reduced by blocking EphA2 in endometrial carcinoma cells or knockout of *EPHA2* gene in renal and colon tumor cells, which indicates the interaction of EphA2 and γδ T cells play an important role in enhancing the susceptibility of γδ T cytotoxic reactivity (36, 37).

## 3 The Characteristics of γδ TCR and the Related Co-Stimulation Signals in γδ T Cells

### 3.1 γδ TCR Signal

Since γδ T cells eliminate tumor cells *via* recognizing a variety of tumor-associated antigens, γδ TCR signals play a key role in regulating γδ T cell activation. Like conventional αβTCR, γδ TCR is a complex of a clonotypic heterodimer TCRδ/TCRγ, two CD3 dimers (CD3δϵ or/and CD3γϵ), and a ζζ dimer (38). The CD3ϵ-deficient patients had complete deficiencies in peripheral T cells, suggesting that the ϵ subunit plays a pivotal role in the αβ T cell development (39). However, some CD3 molecules may play different roles in the functions of γδ T cells. For example, CD3δ^-/-^ mice have normal numbers of γδ T cells (40, 41). In addition, mouse γδ TCRs, which are naturally CD3δ-deficient, can induces calcium mobilization and ERK activation (42). On the contrary, if CD3δ is deficient in human or mice, the development of αβ T cells are failed (40, 41), and did not induce signaling events by the engagement of CD3δ-deficient αβ TCRs (43). Another important CD3 molecule, CD3γ, only blocks, but not significantly impairs the development of γδ T cells in human, as *CD3δ* gene may rescue the γδ T cell development (44). Current researches reported the function of TCR/CD3 complex components in signaling transmitting in αβ T cells, which was applied in engineered αβ T cells and engineered γδ T cells. For example, CD3ζ chain was determined to transmit signals in the absence of CD3 γ, δ, and ϵ in αβ T cells (45), which was widely used to deliver a major activation signal in both αβ T cell and CAR-T cells. In addition, in absence of CD3ζ chain, the CD3 γϵ/δϵ, or CD3ϵ alone were also able to independently activate αβ T cells (46, 47). However, the specific signaling function of TCR/CD3 complex components have not been precisely reported in γδ T cells, which needs to be explored in the future. As a whole, signals transmitted by TCR in αβ T cells and γδ T cells are not always the same. A clinical test of 60 samples from hospitalized and healthy individuals demonstrated that human γδ T cells constitutively expressed higher density of TCR/CD3 complex (2.12 ± 0.33 fold) than that in αβ T cells (48). Furthermore, by analyzing the ability to induce calcium mobilization, ERK activation, and cellular proliferation in mouse γδ T cells, it revealed a superior effect on γδ T cells in the aspect of signal transduction than that in αβ T cells with the same stimulation using immobilized anti-CD3 monoclonal antibody (mAb), which revealed that γδ T cells have a better signal-transducing complex than αβ T cells (42). Interestingly, in steady state, compared with αβ T cells, mouse γδ T cells have higher phosphorylation levels of ERK1/2 and stronger proliferation ability. Therefore, it suggested that γδ T cells possess a more “primed for action” status at baseline even in the absence of any external stimulation (49).

TCR conformation also influences the signal. CD3 conformational change (CD3 CC), which takes advantage of the increased accessibility of a proline-rich sequence (PRS) in the CD3ϵ cytoplasmic tail, was required for T cell activation (50, 51). In αβ T cells, cholesterol bound to the transmembrane region of TCRβ keeps the TCR in a resting and inactive conformation that cannot be phosphorylated by active kinases. Only αβ TCRs that spontaneously detached from cholesterol could switch to the active conformation (termed primed TCRs) and then be phosphorylated (52). Moreover, αβ TCR signaling could be inhibited by cholesterol sulfate, suggesting an important role of cholesterol in the conformation of αβ TCR (53). But γδ TCRs does not bind to cholesterol, accounting for a higher percentage of γδ TCRs in the active conformation compared to αβ TCRs (52). In addition, the CD3 CC in Vγ9Vδ2 T cells induced by anti-CD3ϵ mAb stimulation, which dramatically enhanced target cell lysis of the pancreatic tumor cell line Panc89 (54). The better reactivity of γδ T cells provides a better application of engineered γδ T cell therapy.

### 3.2 Co-Stimulation Molecules

**Figure 2:**
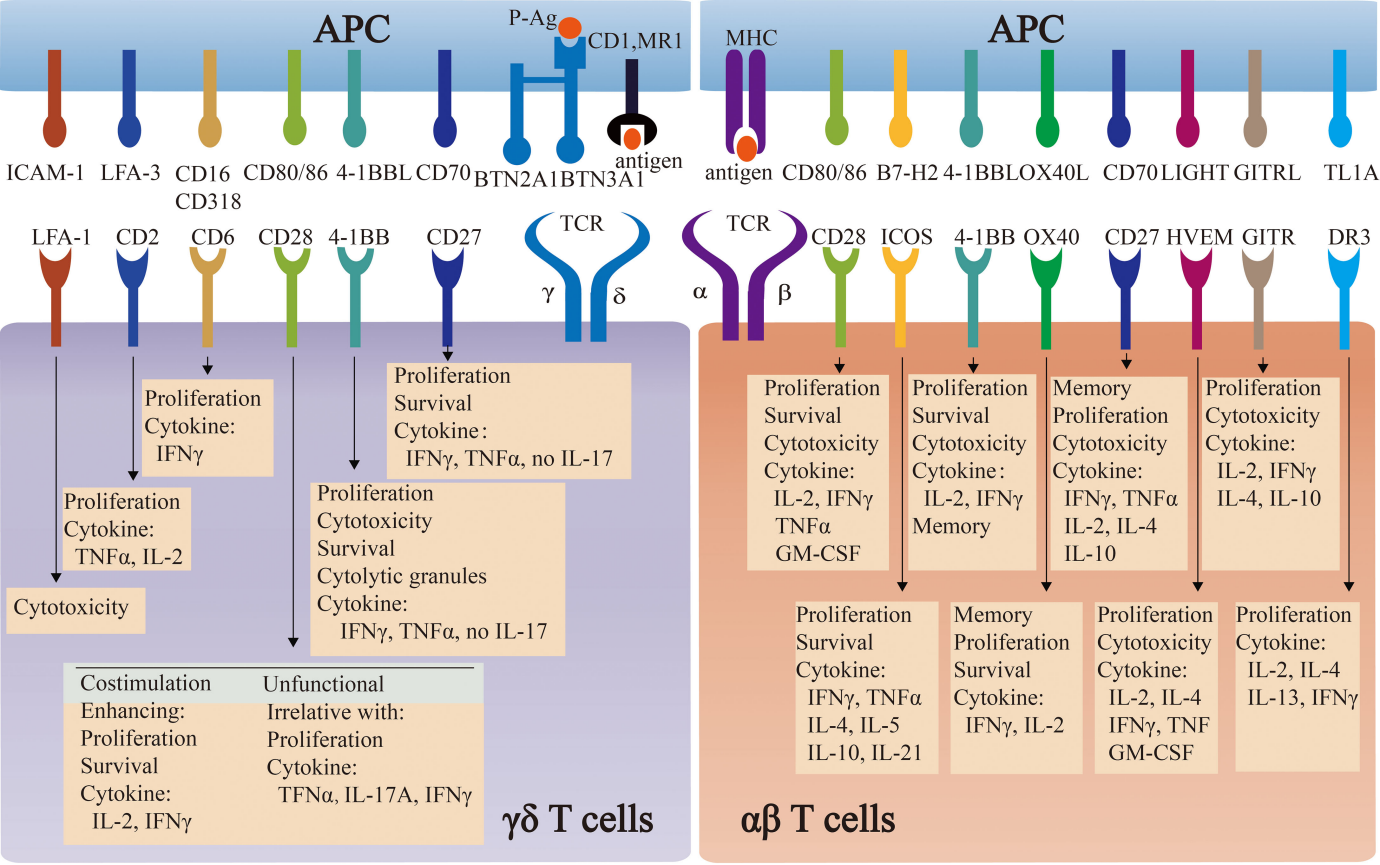
The comparison of co-stimulatory molecules and their induced effector functions between αβ T cells and γδ T cells. Co-stimulatory molecules induce the effector functions of αβ T cells and γδ T cells, by engaging respective ligands and counter-receptors on APCs. The specific functions of some molecules are commonly well-understood in αβ T cells, whereas they are controversial in γδ T cells.

Apart from TCR-dependent stimulation, the co-stimulation signals are also important and widely applied into the 2^nd^ generation of CAR-T therapy. To date, almost all engineered γδ T cells follow the co-stimulation design in αβ T. However, whether these co-stimulation signals are applicable to engineered γδ T cells requires further investigation. The comparison of co-stimulatory molecules and their induced effector functions between αβ T cells and γδ T cells is summarized in **Figure 2**.

#### 3.2.1 CD28

Almost all engineered γδ T cell, especially CAR-γδ T cell, utilize CD28 as a co-stimulation molecule. CD28 is an important co-stimulation molecule that express on most CD4^+^ and half of CD8^+^ αβ T cells. It has been widely accepted that CD28 mediates costimulatory signal to amplify signaling generated by TCRs ligation, promoting proliferation, survival and cytokine production of αβ T cells (55). Thus, CD28 has been widely applied in CAR-αβ T cells to help exert better effect. However, co-stimulatory function of CD28 in γδ T cells is still under debate. Some studies indicated that CD28 functioned as a costimulatory molecule in γδ T cells. CD28^+^ γδ T cells have the better activation, proliferation, survival and production of IL-2 when they were stimulated with anti-CD28 mAb (56–58). It also showed that almost no γδ T cells, especially CD69^+^ γδ T cells, could expand in CD28-deficient malaria mouse model. Along this line, CD28^-/-^ γδ T cells failed to produce cytokines, such as IFNγ and IL-17. In human, the blockage of CD28 ligand led to the impairment in γδ T cell proliferation and survival (56). However, since CD28 signal is extremely important for αβ T cells, including CD4^+^ T help cells, neither the CD28^-/-^ mice infection model nor the CD28 ligand blockade experiment can exclude the possibility that blocking CD28 signal reduced the function of CD4^+^ T help cells, thereby affecting γδ T cells indirectly. On the contrary, some studies disagreed with the co-stimulatory function of CD28 in γδ T cells. Some researchers found that CD28 was not expressed in resting mouse splenic, intestinal intraepithelial, and vaginal γδ T cells, revealing the dispensable role of CD28 in mouse (57, 59, 60). In addition, the proliferation of γδ T was unchanged when they were stimulated with anti-CD3 mAb with or without anti-CD28 mAb (42). Consistently, CD28^+/+^ and CD28^-/-^ mice were revealed to have equivalent increases in the percentage and quantity of the γδ T cells and IL-17A^+^/IFNγ^+^ γδ T cells in a listeria model of Infection (61). In human, although 40-60% freshly isolated human γδ T cells expressed CD28, this subset was diminished to 10% during *in vitro* culture, and even disappeared in long-term culture (62, 63). Moreover, human Vγ9Vδ2 T cells produced TNFα *via* direct TCR-induced p38 kinase and MEK/ERK activation pathway, but irrelative with CD28 (63). As is discussed above, since the co-stimulatory function of CD28 remains controversial in different stimulating conditions or infection models, it is still unclear whether γδ T cell function requires transient or continuous CD28 signals. Comprehensive studies to investigate the role of CD28 signals in γδ T cells are required, which will benefit for the better design of engineered γδ T cells.

#### 3.2.2 4-1BB

4-1BB, also known as CD137, is an inducible T cell costimulatory molecule. It can be detected after stimulation and reaches the peak of expression at 48h in human αβ T cells, and functions as a conventional co-stimulatory molecule (64). 4-1BB has been widely applied in engineered αβ T cells, but not in γδ T cells currently. Many researches pointed that 4-1BB preferred to help expand memory CTLs, up-regulated NKG2D expression and rendered enhanced cytolysis. More importantly, the 4-1BB provided a stronger cytotoxicity than CD28 in some experiments (65). However, no research has specifically evaluated the advantages and disadvantages of 4-1BB in engineered γδ T cells, which raises the question of whether 4-1BB can exert as an efficient co-stimulator in engineered γδ T cells. Existing researches have revealed the co-stimulation function of 4-1BB in γδ T cell in different disease models. For example, 20% 4-1BB^+^ Vγ9Vδ2 T cells were observed in influenza virus infection model, and most importantly, such cell subset showed an enhanced *ex vivo* effector function such as more intensive granule release, more cytokine production (e.g. IFNγ), and superior cytotoxic activity towards virus-infected cells comparing to the 4-1BB^-^ counterparts. Furthermore, the co-stimulation effect of 4-1BB was determined to induce better proliferation and enhance the survival of Vγ9Vδ2 T cells (66). On the other hand, in influenza virus infection mouse model, the transfer of 4-1BB^+^ γδ T cells was beneficial to maintain the body weight, enhance the survival rate, and reduce virus titers. With the co-stimulated of 4-1BB, only γδ T cells, but not other subsets of PBMCs, had improved therapeutic outcome in this disease model (66). In addition to influenza infection model, *Listeria Monocytogenes* infected mouse model indicated that compared to γδ T cells without 4-1BB stimulation, γδ T cells with 4-1BB stimulation showed the decreased bacterial load *in vivo* and enhanced survival. To be more specific, anti-4-1BB treatment in adoptive γδ T cell treatment, rather than adoptive αβ T cell treatment, significantly increased the cytokine production such as IFNγ and TNFα, and the augmented number of γδ T cells (67).

#### 3.2.3 CD27

CD27 is also a stimulatory molecule in αβ T cells, which interacts with CD70 and induces the activation, proliferation, and survival of αβ T cells (68). It has been applied in CAR-αβ T which can promote the proliferation, anti-tumor effect, and survival both *in vitro* and *in vivo* (69). However, it has not been applied in engineered γδ T cells. CD27 has been found to be widely expressed in γδ T cells. It is expressed in 70-90% of γδ T cells in mouse spleen and lymph nodes (70), 81% of activated Vγ9^+^ T cells, and even some Vδ1^+^ T cells in peripheral blood in human (71). Many researches have showed its co-stimulation function as it can promote proliferation, survival and cytokine production in γδ T cells (56, 71). Interestingly, CD27 was used to distinguish mouse γδ T cells with different cytokine production. In this case, CD27^-^ γδ T cells produce IL-17, whereas CD27^+^ γδ T cells produce IFNγ. In addition, 90% IFNγ and 70% TNFα-producing cells were CD27^+^ γδ T cells in naïve and malaria-infected mice (70). Apart from these *in vitro* researches, γδ T cells failed to expand in CD27 deficient mice when infected with MuHV-4, compared to that in WT mice. Moreover, the deficiency of CD27 related to the anergy of IFNγ production (72). In human, comparing to CD27^-^ γδ T cells, CD27^+^ Vγ9Vδ2 T cells showed higher level of proliferation and up-regulation of BCL2A1 gene after being cultured with HDMAPP. Vγ9Vδ2 T cells had a stronger ability of proliferation and IFNγ, LT-α secretion under sCD70 stimulation, and CD70 blockade prevented efficient expansion of Vγ9Vδ2 T cells and reduced production of TNFα and LT-α (71). As summarized, CD27 may be a potential co-simulation molecule that can be applied in engineered γδ T cells.

#### 3.2.4 Potential Co-Stimulation Molecules

Recent researches have revealed some other potential co-stimulation molecules. For example, CD6 (ligands to CD166 and CD318), a costimulatory receptor, is expressed on virtually all T cells, especially activated human γδ T cells (73–75). After stimulated by CD166, human Vδ2^+^ T cells showed increased proliferative capability and IFNγ production. In addition, both CD6 and CD166 were observed to locate at center of synapses in activation process (75). In αβ T cells, a CAR with CD6 showed increased release of IFNγ and enhanced anti-tumor effect when compared with the CAR without CD6 (76). However, CD6 has not been applied in engineered γδ T cell therapy. In addition to CD6, CD2 and LFA1, as adhesion molecules, also have costimulatory function in activated αβ T cells (77, 78). Ligation of CD2 and its ligand was applied in first-generation CD19-specific CAR to drives IL-2 production (79).There are several researches about its costimulatory function in γδ T cells. The stimulation by anti-CD2 mAb promotes IL-2 secretion and/or proliferation of γδ T cells (80). Correspondingly, the blockage of CD2 or LFA1 inhibited the effector function, especially reduced TNFα production, of Vδ2^−^ T cells (34). However, LFA1 and CD2 signals affected the function of Vγ9Vδ2 T cells differently. CD2 blockade strongly inhibited proliferation of γδ T cells and release of TNFα/IL-2, but had no effect on the lytic activity of γδ T cells, whereas LFA-1 blockade had no effect on cell proliferation and cytokine production, but could effectively inhibited target cell lysis (81). Consequently, CD2/LFA1 co-stimulation may differently influence the effector function of engineered γδ T cells.

## 4 The Characteristics of NK Cell Receptor Signals in γδ T Cells

The expression of a variety of NK cell receptors, including NKRs and NCRs, is an important feature of γδ T cells, which endows γδ T cells innate immune characteristics like NK cells. Also, the two kinds of lymphocytes share similar characteristics in the perspective of immune responses. Compared to αβ T cells, involvement in innate immune reaction is beneficial for γδ T cells to recognize a more extended spectrum of antigens on tumor cells, reduce the risk of tumor immune escape by losing single tumor-associated antigen, and provide available chances for novel immunotherapies for cancers that lack tumor specific antigens.

### 4.1 Natural Killer Group 2D (NKG2D)

As one of the most important receptors, NKG2D is a C-type, lectic-like, type II transmembrane glycoprotein, which is expressed on NK cells, γδ T cells and some narrowed subsets of αβ T cells (82, 83). In human peripheral blood, almost all γδ T cells expressed NKG2D, but compared with NK cells, the expression level of NKG2D is about 10-times lower (82). In addition, the intestinal intraepithelial γδ T cells originally express relatively low level of NKG2D. Interestingly, the expression of NKG2D can be upregulated in response to IL-15 stimulation or 4-1BB signals (84).

#### 4.1.1 NKG2D Recognition

Like NK cells, NKG2D on γδ T cells can also recognize ligands including MHC class I-like molecules [e.g. MHC I chain-related molecules A and B (MICA/B) and UL16-binding protein (ULBP1-6)] in human, and retinoic acid early transcripts (Rae1) α-ϵ, murine UL16-binding protein-like transcript 1 MULT1, H60a, H60b, and H60c in mouse. These ligands can be induced in infected and oncogenic transformed cells (85, 86). Therefore, NKG2D is frequently involved in the tumor cell recognition, induces cytokine release, and triggers degranulation. NKG2D is reported to trigger cytotoxicity of γδ T cells against tumor cells and bacterial or virus-infected cells in a NK-like and TCR-independent manner (87–91). Stimulated by anti-NKG2D mAb or NKG2D ligand protein (NKG2DL), human Vγ9Vδ2 T cells and mouse dendritic epidermal T cells (DETCs) released cytotoxic granules and cytokines such as TNFα. The blockade of NKG2D completely abolished such cytotoxicity to tumor cells induced by Vγ9Vδ2 T cells (83, 87, 91).

#### 4.1.2 NKG2D Signal

The pivotal role of NKG2D in γδ T cells has attracted researchers to explore the underlying signaling molecules and specific signaling pathways. The NKG2D signals are much similar between γδ T cells and NK cells. In NK cells, NKG2D is associated with adapter molecules DAP10 to transmit signals in PI3K or Vav/SOS signaling pathway to trigger cytotoxicity, but without IFNγ production. Alternatively, NKG2D connects to DAP12 to recruit Syk and ZAP70 to downstream signaling events to trigger cytotoxicity, along with the secretion of IFNγ in mice (92–96). In γδ T cells, NKG2D has been observed to act in a PI3K-dependent signaling pathway that responds to target cells in a TCR-independent manner (88, 91, 97). DAP10, rather than DAP12, was reported to strongly express in resting and activated human Vγ9Vδ2 T cells (82, 96), while DAP10/DAP12 constitutively expressed in mouse DETCs (83). Human Vγ9Vδ2 T cells stimulated by ULBP proteins could produce IFNγ, TNFα, and released cytolytic granules usually accompanying PKB (a PI3K kinase substrate) phosphorylation (88, 97). The knockdown of either DAP10 or NKG2D in Vγ9Vδ2 T cells showed the similar impaired anti-bacterial effect, when cocultured with infected macrophages. This indicated that DAP10 is involved in NKG2D signaling during bacterial infection (88). In mouse DETCs, NKG2D could trigger a PI3K-dependent signaling pathway by DAP10 to increase phosphorylation of Akt, trigger degranulation and induce cytotoxicity, which could be completely inhibited by PI3K inhibitor. In addition, in the absence of both NKG2D-S-DAP12 (a shorter protein isoform that is produced by alternative splicing of *killer cell lectin-like receptor K1(Klrk1)*) and TCR signals, only NKG2D/DAP10 signals through the PI3K/Grb2/Vav1 pathway is sufficient to trigger cytotoxicity of DETCs against target cells (91). Of note, although γδ T cells could produce IFNγ and were cytotoxic under the stimulation of anti-NKG2D mAb (83), these cells failed to produce IFNγ, TNFα, IL-13 and induced Syk/ZAP70 activation when stimulated by recombinant NKG2DL protein. It indicated that NKG2DLs may not be able to engage enough activation of NKG2D/DAP12 signaling, which might be weaker than NKG2D/DAP10 on DETCs in the aspects of triggering Syk/ZAP70 signaling (91).

#### 4.1.3 Signal Difference Between NKG2D and TCR in γδ T Cells

Apart from the signaling mechanism, another interesting question is the different activation level through NKG2D and TCR in activating γδ T cells. Some experiments compared effector function in cytokine production, degranulation, killing ability induced by γδ T cells with specific TCR stimulation to that with NKG2D stimulation. For example, comparison of cytokines production in different stimulation groups, Vγ9Vδ2 T cells induced the similar level of TNFα when stimulated by anti-NKG2D mAb or recombinant MICA-Fc or Daudi cells plus IPP, although which is weaker than that of stimulation with anti-CD3ϵ mAb (87). Furthermore, by stimulating NKG2D or TCRs pathways with antibodies or cell lines respectively, Vγ9Vδ2 T cells showed the similar level of degranulation, IFNγ production and cytotoxicity (87). In the real situation, tumor cells can express ligands that could bind both NKG2D and γδ TCRs. Therefore, researchers conducted inhibition experiment to compare the contribution of NKG2D and TCR to the cytotoxicity of γδ T cells. The inhibitory effect of TCR signal blocking on Vγ9Vδ2 T cell cytotoxicity was much stronger than that of NKG2D signal blocking (87, 97). However, TCR signal or NKG2D signal blockade had similar inhibitory effects on cytotoxicity of DETCs, indicating that the signaling pathways in human and mouse γδ T cells are different (91). Interestingly, some ligands that can be recognized by both γδ TCRs and NKG2D, such as ULBP4, can be killed by Vγ9Vδ2 T cells in both TCRs-based and NKG2D-based activation pathways. Blocking one of these pathways could only induce minor inhibitory effect on degranulation of Vγ9Vδ2 T cells and cytotoxicity to EL4-ULBP4^+^ cells, but almost completely inhibited IFNγ production by Vγ9Vδ2 T cells. However, blocking both of these pathways could significantly reduce the cytotoxicity of Vγ9Vδ2 T cells (97). In addition, NKG2D signal in γδ T cells could enhance TCR-dependent signals, which increased cytokine production and cytotoxicity of γδ T cells, and extended survival of γδ T cells (98–101). For calcium response, compared with αβ T cells and iNKT cells, which showed a strong and rapid TCR-induced Ca^2+^ response, Vγ9Vδ2 T cells showed a delayed and sustained Ca^2+^ response. However, when NKG2D signal was simultaneously activated, Ca^2+^ responses in γδ T cells induced by TCR signal could be accelerated. Besides, NKG2D signal alone could not induce significant Ca^2+^ activation signals, indicating NKG2D signal could enhance TCR signal in γδ T cells. Furthermore, PKCθ were found to play an important role in the NKG2D mediated costimulatory function. Vγ9Vδ2 T cells have significantly improved cytolytic ability to tumor cells with NKG2D signal, which could be blocked by PKCθ inhibitor. It is worth noting that PKCθ inhibitor could inhibit the acceleration of Ca^2+^ response induced by NKG2D, indicating that NKG2D signal could shape Ca^2+^ response and potentiate antitumor CTL activity of Vγ9Vδ2 T cells in a PKCθ-dependent manner (99). Taken together, regulation of NKG2D on DAP10 and/or DAP12 signals alone or together with TCR signals should be carefully designed for the application of engineered γδ T cell therapies, especially for the characteristic of cytotoxicity, proliferation, exhaustion, memory and cytokines production of engineered γδ T cells.

### 4.2 Natural Cytotoxicity Receptors (NCRs)

Like NKRs, NCRs are mostly detected on Vδ1 population, and can activate γδ T cells by recognizing ligands on tumor cells (102–104). They exert potent anti-tumor activities in a TCR-independent way (103–105), and sometimes enhance effector function of γδ T cells (106). Similar to NKG2D, NCRs ligated with adaptor proteins, such as CD3ζ, FcRγ and DAP12, to transmit intracellular activating signaling in NK cells. Similarly, NKp44 is detected to couple with DAP12 in stimulated human γδ T cells (106). But the specific function of these adapters associating with NCRs in γδ T cells remains unclear and needs further exploration.

**Figure 3:**
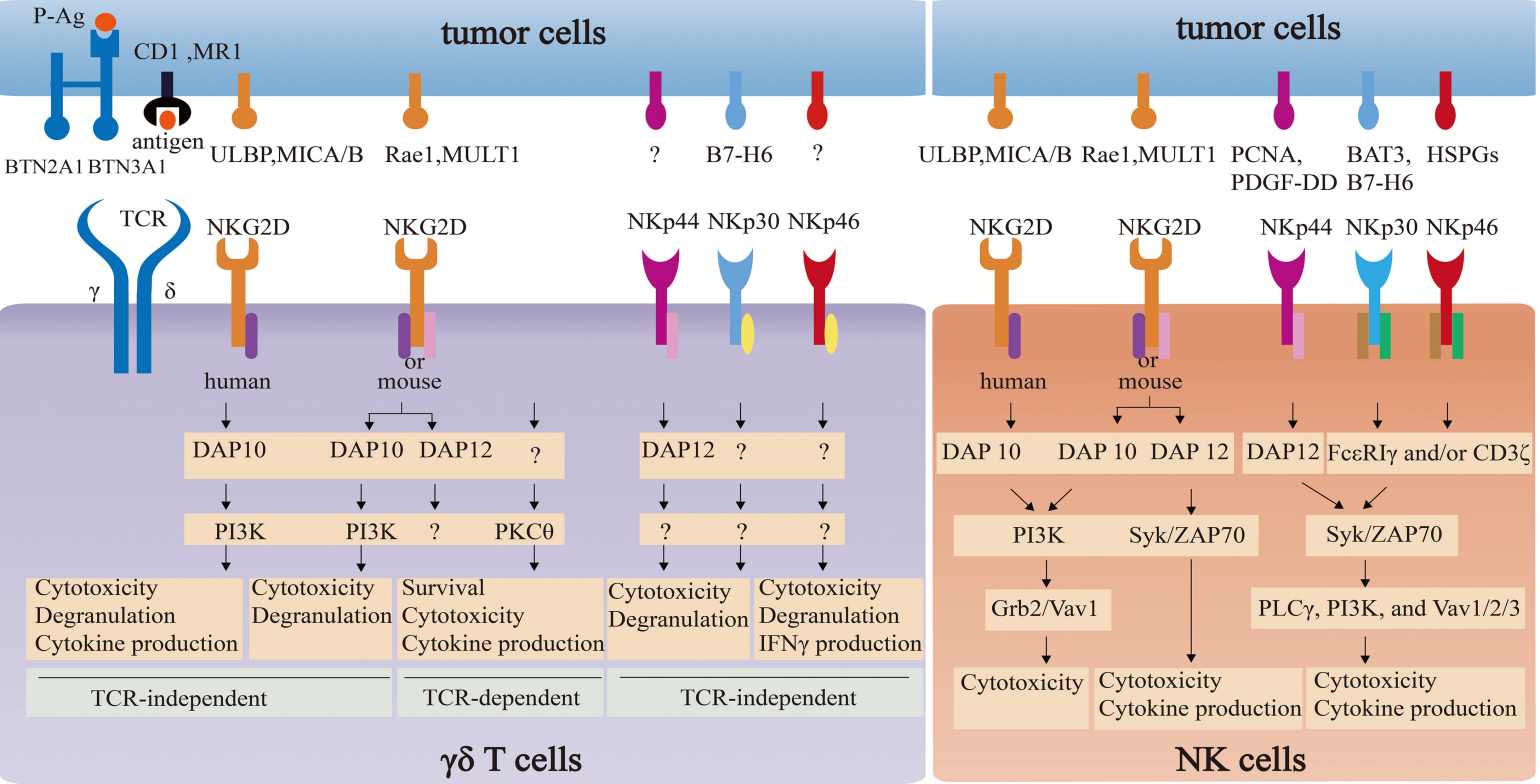
The comparison of NKG2D and NCR signaling pathways between γδ T cells and NK cells. NKG2D in NK cells associates with adapter DAP10 (PI3K and Grb2-Vav1 signaling) or DAP12 (Syk/ZAP70 signaling) to directly induce cytotoxicity and/or cytokine release. NKG2D in γδ T cells not only directly triggers cytotoxicity *via* PI3K-dependent pathway by coupling with DAP10, but also enhances the effector function in a TCR-independent way *via* PKCθ. However, the impact of NKG2D-DAP12 complex on the function on γδ T cells remain elusive. NCRs in NK cells and γδ T cells can induce cytotoxicity activity. However, signaling pathways of NCRs-ligands in NK cells are well-understood, which in γδ T cells remain unclear.

The comparison of NKG2D and NCR signaling pathways between γδ T cells and NK cells is summarized in **Figure 3**.

## 5 Other Receptors Delivering Signals in γδ T Cells

γδ T cells also express cytokine receptors, like IL-2Rβγ, IL-18R, IL-7Rα, IL23R (107–111), which can deliver activated signals by binding to interleukins. Stimulation of these cytokine receptors can not only enhance the effect function of γδ T cells, but also directly trigger the activation of γδ T cells even in the absence of TCR signal. For example, after initial stimulation by P-Ag or γδ TCR antibody, IL-15, IL-12, IL-2, IL-18, IL-33 and IL-7 could additionally enhance the proliferation, cytokine production, cytotoxic effect of γδ T cells (112–118). Furthermore, some cytokines alone or combination, such as IL-15, IL-2, IL-12, IL-18, IL-7, IL-1 and IL-23, were found to induce proliferation, cytokine production and killing ability in the absence of TCR signal (107, 110, 111, 115). Besides, γδ T-induced effector molecules were impacted by cytokines. IL-2, IL-12, IL-18, IL-15 and IL-21 were found to promote IFN-γ-production of γδ T cell (113, 115, 119, 120), whereas IL-17-production of γδ T cell was driven by IL-1, IL-23 and IL-7 (111, 118, 121). Interestingly, IL-18 could replace IL-1β and cooperate with IL-23 to induce IL-17 production in γδ T cells (108). In addition, toll-like receptors (TLRs) were also reported to deliver activated signals in γδ T cells. The simultaneous stimulation of TLRs (e.g. TLR1/2/6, 3, and 5) and TCR significantly enhanced the activation and effect function of γδ T cells. Furthermore, γδ T cells can also directly respond to TLR2 ligands to act effect function in a TCR-independent manner (122).

## 6 Application of γδ T Cells in Engineered T Cell Therapies

Although γδ T cells have limited ability to expand and proliferate *in vivo*, which may affect the antitumor efficacy of γδ T cells, γδ T cells remain good candidates for engineered therapies with many advantages. Firstly, since engineered γδ T cells can exploit the endogenous receptors (TCRs and innate immune receptors) and engineered receptors, the current CAR-γδ T therapies induce a significantly stronger potential to kill targeted cells and cytokine production, which contributes to more significant reductions of tumor burden and suppression of tumor growth compared with γδ T cells (123–126). These endogenous receptors enable γδ T cells not only to recognize a myriad of tumor-specific or associated ligands as described above, but also to prevent tumor escape caused by antigen loss or downregulation (127). In this scenario, the downregulation of MHC-I in tumors helps tumor cells to escape surveillance of αβ T cells, but it does not inhibit non-MHC-restrict γδ T cell activation and even enhances the consecutive γδ T cell activation (128). Indeed, compared with CAR-αβ T cells, CAR-γδ T cells targeting CD19 or melanoma cell surface chondroitin sulfate proteoglycan (MSCP) showed a significantly higher cytotoxicity against tumor associated antigen (TAA) negative target cells, or β2-microglobuline-deficient Daudi cells that lacks the expression of MHC-I (125, 129). Secondly, activated human Vδ2^+^ T cells can present the characteristics of professional antigen-presenting cells like dendritic cells, which can take up, process, and present soluble antigens to αβ T cells. HLA-A0201^+^V2δ^+^GD2-CAR-γδ T cells can present the epitopes of melanoma antigen recognized by T cells 1 (MART-1) to αβ T cells to promote expansion and cytotoxicity (130). Thirdly, since allogeneic αβ T cell therapies have side-effects of host-versus-graft activities (HVGA) and graft-versus-host disease (GVHD), current engineered αβ T cell products are individualized and have many limitations, such as high cost, time consuming, and unstable quality or quantity of T cells (131). However, engineered γδ T cells with MHC-unrestricted recognition pattern can avoid of GVHD, which makes it possible for engineered γδ T cells to become universal cell products to circumvent many disadvantages of above-mentioned individualized CAR-T cell products. Many clinical cases and trials were trying to assess the safety of allogeneic γδ-TCR T cell therapies to confirm the absence of GVHD by γδ T cells. For example, after receiving allogeneic γδ T cell immunotherapy, a patient with cholangiocarcinoma had improved peripheral immune function, reduced tumor activity, and prolonged life span, and more importantly, without side-effects (132), indicating the safety of γδ T cells and its potential to be universal. Besides, 3 clinical trials (NCT04107142, NCT04735471, NCT04911478) were conducted to evaluate the safety and tolerability of allogeneic CAR-γδ T cells targeting NKG2D ligand (NKG2DL) and CD20. However, HVGA and the persistence remained to be the challenge for engineered γδ T cell products. Taken together, engineered γδ T cells take advantage of recognizing antigens by endogenous receptors as well as engineered receptors, processing and presenting antigens to activate αβ T cells and avoiding GVHD. To sum, γδ T cells can be a more efficient and wider-applied antitumor candidate to produce engineered products.

## 6.1 CAR Transfer to γδ T Cells

CAR-αβ T therapy has shown unprecedented success in hematologic malignancies, but poor efficacy in solid tumors. Many studies found higher infiltration of γδ T cells in solid tumors than that of αβ T cells, and the frequency of infiltrated γδ T cells in solid tumors positively correlated with prognosis (133–135), indicating a promising application of CAR-γδ T cells in solid tumors. Thus, CAR-γδ T cells have been designed to target many solid tumor antigens, such as disialoganglioside 2 (GD2) on neuroblastoma and Ewing sarcoma (136), melanoma chondroitin sulfate proteoglycan (MCSP) on melanoma lesions (137), original or glycosylated Mucin 1 (MUC1) on breast cancer, head and neck squamous cell carcinoma (138, 139). Current ongoing clinical trials involving engineered γδ T products are summarized in **Table 1**.

**Table 1:** Current ongoing clinical trials of engineered γδ T products.

Clinical Trials/Netherlands Trials Identifier	Phase	Disease	Interventions	Source	γδ T subset
NCT04735471	I	B Cell Malignancies	CD20-CAR expressed on γδ T cells	allogeneic	Vδ1 γδ T-cell
NCT04107142	I	solid tumor.	NKG2D-CAR expressed on γδ T cells	haploidentical/allogeneic	unmentioned
NCT04702841	I	T cell-derived malignant tumors	CD7-CAR expressed on γδ T cells	unknown	unmentioned
NCT03885076	unknown	AML	CD33-CAR expressed on γδ T cells	autogenetic	Vδ2 γδ T-cell
NCT04796441	Not Applicable	AML	CD19-CAR expressed on γδ T cells	allogeneic	unmentioned
NCT02656147	I	Leukemia Lymphoma	CD19-CAR expressed on γδ T cells	allogeneic	unmentioned
NL6357	I	r/r AML, high-risk MDS or MM	a defined γδ T cell receptor expressed on αβ T cells	autologous	/

However, current CAR-γδ T cells fails to show better efficacy of tumor immunotherapy than CAR-αβ T cells. One of reasons is the design of intracellular signaling domain of CAR-γδ T cells is less optimized. The intracellular signaling domains applied in CAR-γδ T cells are almost as same as what used in CAR-αβ T cells. Indeed, CAR-γδ T cells are reported to have a significant effector function against tumor cells. But it is controversial in different studies comparing CAR-γδ T cells with CAR-αβ T cells, particularly in solid and hematologic tumors. Meir Rozenbaum et al. pointed the superiority of CAR-αβ T cells in leukemia *in vivo*. To be more specific, treatment with CAR-γδ T or CAR-αβ T cells led to a respective 5% and 0.1% tumor cell residue in the bone marrow of mice, demonstrating the higher load of leukemia cells in recipients of CAR-γδ T cells compared to the CAR-αβ T treated mice (125). This phenomenon suggested that CAR-γδ T cells with suboptimal design have lower efficiency to eliminate tumor cells than that by CAR-αβ T cells. Furthermore, recent study reported the persistence of CAR-Vγ9Vδ2 T cells was worse than that of CAR-αβ T cells. While CAR-αβ T cells still effectively eliminate all the tumor cells in the fourth round of tumor stimulation, CAR-Vγ9Vδ2 T cells almost lost their cytotoxicity. Fortunately, the cytotoxicity of γδ T cells can be restored by the addition of IL-2 (126). Although compared with CAR-αβ T cells, CAR-Vδ1 and Vδ2 T cells secreted higher levels of granzyme B and cytokines, and exhibited similar or stronger cytotoxicity against some kind of solid tumors *in vitro* (126), the specific effector function against solid tumor *in vivo* should be comprehensively investigated in the future. Therefore, it is extremely important to investigate the optimal use of activation signals for CAR-γδ T cells. Recent studies have made some modifications to simultaneously take advantage of the natural endogenous signal properties of γδ T cells. For example, DAP10 was used in engineered γδ T cells and engaged in the antitumor response. Except for the signal induced by TCRs, GD2-DAP10 CAR transferred γδ T cells used the solitary endodomain derived from the NKG2D adaptor DAP10 to mimic NKG2D co-stimulation, which induced significant cytokine production and equivalent killing as CD28-CD3ζ-CAR-γδ T cells against GD2^+^ Neuroblastoma and Ewing Sarcoma (140). Interestingly, this example also promoted the utilize of “AND gate” system in engineered γδ T cells to minimize on-target off-tumor toxicity. It was only activated in presence of antigen through γδ TCR and GD2, whereas only GD2 could activate CD28-CD3ζ-CAR-γδ T cells (140).

### 6.2 αβ TCR Transfer to γδ T Cells

Engineered γδ T cells not only included CAR-γδ T cells, but also TCR-γδ T cells. For example, αβ TCRs were reported to be transferred to γδ T cells, making αβ TCR-γδ T cells sensitive to tumor cells with antigen-negative or tumor escape variants with MHC-downregulating. γδ T cells which expressed an HLA-A*0101 restricted αβ TCR targeting the adenovirus hexon protein of HAdV-species C, released more IFNγ and TNFα than CD8^+^ αβ T cells with the same αβ TCR, and had comparable cytotoxicity against adenovirus-infected dendritic cells (141). Interestingly, while most γδ T cells lack the expression of the co-receptors CD4 or CD8, some researches transferred the co-receptors along with αβ TCRs to γδ T cells and found the enhanced specific functional activity. Comparing to HA-2-TCR-γδ T cells without the additional transfer of CD8, co-transferring of CD8 and HA-2-TCR to γδ T cells significantly increased IFN-γ and IL-4 production and exerted more efficient cytotoxicity against the HA-2-expressing CML and AML cells (142). In addition, transferring αβ TCRs that recognized the same antigen as endogenous γδ TCRs could improve TCR-γδ T cell antigen recognition and cytotoxicity efficiency. For example, transferring αβ TCRs derived from invariant natural killer T (iNKT) cells, which recognized glycolipid antigens presented by CD1d, the TCR-γδ T cells were found to respond to CD1d *via* both endogenous γδ TCRs and transferred αβ TCRs, and had increasing antitumor effect against the CD1d positive leukemia cell line K562 (143). Of note, the transfer of αβ TCRs to γδ T cells did not show any mispairing of endogenous and transgenic TCRs (144), which significantly avoided autoimmunity (145, 146). Along this line, in order to obtain better anti-tumor efficacy, CAR-γδ T cells or TCR-γδ T cells can be designed so that endogenous γδ TCR and engineered CAR/TCR can recognize the same antigen, such as CAR-γδ T cell targeting NKG2DL or BTN3A, TCR-γδ T cell targeting HLA-A24, HLA-B27 and HLA-A2, all of which can be investigated in the future.

### 6.3 γδ TCRs Transfer to αβ T Cells

γδ TCRs transferred αβ T cells was also used to overcome the deficiency of cytotoxicity of particular types of HLA-restricted αβ T cells. This design has several advantages. Firstly, the γδ TCRs could target a broad range of solid and hematological tumors in MHC-independent manner. Secondly, compared with γδ T cells, the mechanism of effects and memory functions of CD4^+^ and CD8^+^ αβ T cells are better understood *in vivo* (7). Thirdly, this strategy can avoid the activity of inhibitory receptors like KIRs on γδ T cells. Indeed, αβ T cells expressing the Vγ9Vδ2 TCR clone G115 displayed a γδ T cell-like effector function, such as cytotoxicity against the Daudi cell line, cytokine release, enhanced cytotoxicity using amino-bisphosphonates, and the ability to induce dendritic cell maturation. Surprisingly, endogenous αβ TCRs were down-regulated after the transduction of γδ TCRs, leading to a lack of allo-reactive response (147). Besides, several types of tumor specific CDR3δ-grafted γδ TCRs were also used to modify αβ T cells and exhibited significant antitumor effects (148, 149). Moreover, a novel antibody-TCR (Ab-TCR) modified αβ T cells, combining Fab-based antigen recognition with γδ TCR signaling, showed a similar cytotoxicity and a less cytokine release comparing with CD28/CD3ζ CAR-T cells (150). Recently, TEG001, an engineered αβ T products expressing a defined γδ TCR, was proved to be safe and efficient against tumor models *in vivo* (151), and currently was applied in a first-in-human clinical study (NL6357).

## 7 Conclusion

Currently, increasing studies have confirmed the anti-tumor activities of γδ T cells in targeting various malignancies with their innate and adaptive immunities, which brings hopes to the engineered γδ T cells in cancer treatment. However, current engineered γδ T products almost copy the structure of engineered αβ T cells, owing to the ignore of the specific activating mechanism of γδ T cells. As discussed above, we detailed the activating and stimulating modes of γδ T cells *via* TCR signal, some important costimulatory signals, and innate signals from NK receptors, which were summarized in **Figure 1**. Furthermore, current engineered γδ T products and their characteristics are also depicted. Taken the basics of γδ T cells in previous sections together, this review will shed light on the optimal design of engineered γδ T cell to improve its efficacy. However, there are still numerous problems to be solved. More studies are supposed to be conducted to describe the specific activating mechanism of γδ T cells, which can be applied in engineered γδ T products.

## Author Contributions

RD and YZ drafted the manuscript. XZ and HX take the primary responsibility for this paper as the corresponding authors. All authors contributed to the article and approved the submitted version.

## Funding

This work was supported by the National Natural Science Foundation of China (No. 31870899 and 32070899 to XZ, No. 81870136 and 82170141 to HX), Natural Science Foundation of Zhejiang Province (No. LXZ22H080001), and the Independent Task of State Key Laboratory for Diagnosis and Treatment of Infectious Diseases (No.2022zz16).

## Conflict of Interest

The authors declare that the research was conducted in the absence of any commercial or financial relationships that could be construed as a potential conflict of interest.

## Publisher’s Note

All claims expressed in this article are solely those of the authors and do not necessarily represent those of their affiliated organizations, or those of the publisher, the editors and the reviewers. Any product that may be evaluated in this article, or claim that may be made by its manufacturer, is not guaranteed or endorsed by the publisher.

## Abbreviations

ACT, Adoptive cell therapy; AMPK, AMP-activated protein kinase; BTN2A1, Butyrophilin 2A1; BTN3A1, Butyrophilin 3A1; CAR, chimeric antigen receptor; CD3 CC, CD3 conformational change; DETCs, dendritic epidermal T cells; EphA2, ephrin type-A receptor 2; FDA, Food and Drug Administration; FPPS, farnesyl-diphosphate-synthase; GD2, disialoganglioside 2; GVHD, graft-versus-host disease; HVGA, host-versus-graft activities; iNKT, invariant natural killer T; IPP, Isopentenyl pyrophosphate; Klrk1, killer cell lectin-like receptor K1; MART-1, melanoma antigen recognized by T cells 1; MHC, major histocompatibility complex; MICA/B, MHC I chain-related molecules A and B; MR1, MHC-related protein 1; MSCP, melanoma cell surface chondroitin sulfate proteoglycan; MUC1, Mucin 1; NCRs, NK cytotoxicity receptors; NKG2D, Natural killer group 2D; NKG2DL, NKG2D ligand; NKRs, NK cell receptors; P-Ag, phosphoantigens; RAG, recombination activating gene; PRS, proline-rich sequence; Rae1, retinoic acid early transcripts-1; RCC, renal cell carcinoma; scFv, single-chain fragment variable; TAA, tumor associated antigen; TCR, T cell receptors; ULBP, UL16-binding protein; ZOL, zoledronate.
